# Investigating the effects of antipsychotic drugs as a treatment for improving the activity of the *unc-33* /Dpysl2 gene in *C. elegans*

**DOI:** 10.17912/micropub.biology.001063

**Published:** 2024-07-31

**Authors:** Maria C Miranda, Jessica Hyde, Kaylee Salazar, Balyssa Bell, Andrea Holgado

**Affiliations:** 1 St. Edward's University, Austin, Texas, United States

## Abstract

Prenatal stress is hypothesized to contribute to the development of schizophrenia. Lee and colleagues determined that prenatal stress in rats decreases levels of Dpysl2, which is found to be inactivated in schizophrenic patients. UNC-33 , the homolog to Dpysl2 in *C. elegans* , is important for axonal outgrowth and synapse formation. Herein, we study the effects of antipsychotic drugs on developing *C.elegans* exposed to stress through high temperatures. Results indicate that the *unc-33* promoter was not impacted by antipsychotic drug treatment, but the lifespan was decreased.

**Figure 1.**
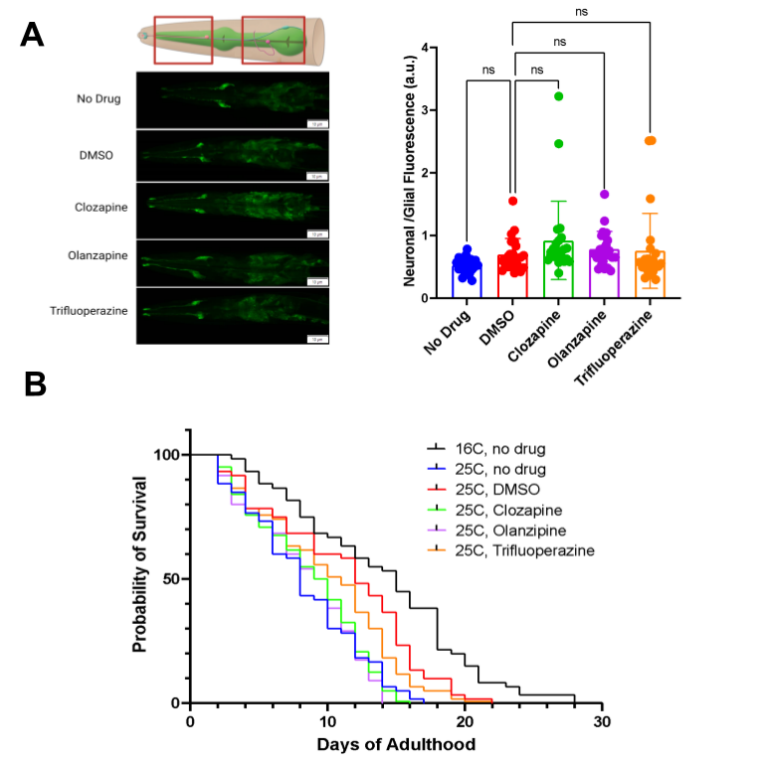
Figure 1: **A: *The neuronal to glial ratio of GFP driven by the unc-33 promoter was not impacted by the treatment with antipsychotic drugs.*** Quantification of the neuronal to glial ratio of GFP driven by the *unc-33* promoter in control groups (no drug and DMSO) and experimental groups (clozapine [160 μM], olanzapine [160 μM], and trifluoperazine [80 μM]). Plotted is the mean ± standard deviation, n=24 nematodes. Kruskal-Wallis test and the post-hoc Dunn's test for multiple comparisons were used for statistical analyses. ns= not significant. Red rectangles denote regions of interest (ROIs). **B: *Antipsychotic drugs decrease the lifespan of animals exposed to stress.*** Lifespan analysis of a transgenic strain expressing GFP driven by the *unc-33* promoter in the presence or absence of antipsychotic drugs. A Kaplan-Meier test followed by the Log-rank Mantel-Cox test denotes that the temperature of 25°C negatively impacts the lifespan of nematodes (****, p<0.0001). Treatment with, antipsychotic drugs clozapine (160 μM) in DMSO and olanzapine (160 μM) in DMSO decreased lifespan, while trifluoperazine (80 μM) in DMSO had no significant effect on the lifespan at 25℃ (p=0.316, p=0.235, and p=0.819, respectively) when compared to worms treated with DMSO alone. Moreover, the longevity of nematodes treated with no drug at 25°C was indistinguishable from the one quantified for nematodes exposed to clozapine in DMSO and olanzapine in DMSO, but was significantly different from those treated with DMSO. Percent survival was derived from observing 60 nematodes per condition from three independent populations of 20 nematodes.

## Description

Schizophrenia is a disease of complex etiology that includes factors such as genetic predisposition and environmental conditions; due to a limited understanding of causality as well as variable presentation of symptoms and deficits, the diagnosis of schizophrenia is often challenging. Schizophrenia is typically characterized by positive symptoms, defined as experiencing things that are not present, usually hallucinations and delusions. Additionally, negative symptoms, lacking something that used to be present, such as motivation, isolation from others, and difficulty expressing emotions, are often experienced by schizophrenia patients. Finally, cognitive symptoms are also hallmarks of the disease, including deficits in executive function, poor memory, and difficulty with attention (Mueser and McGurk 2004) .

A combination of genetic and environmental factors, such as the disruption to the mTOR signaling pathway and prenatal stress, are hypothesized to lead to the development of schizophrenia. Historical records indicate that the development of schizophrenia can be linked to exposure to prenatal stress. For example, the Dutch Hunger Winter, occurring from late 1944 to early 1945, led to a significant increase in the development of schizophrenia in babies born from mothers exposed to severe food deprivation during their first trimester of pregnancy (Susser and Lin 1992; Schulz 2010) . Epidemiological analysis of the Great Chinese Famine also supported the link between food deprivation, prenatal stress, and increased development of schizophrenia (Wang and Zhang 2017) .

To study the molecular mechanisms underlying this link, Lee and colleagues designed experiments in which they subjected pregnant rats to stress such as overnight food deprivation, forced swim tests, reversing light-dark cycles, exposure to cold environments, and overcrowded enclosures. Analysis of pups born of mothers exposed to these stressful conditions showed that the offspring presented schizophrenia-like behaviors, including excessive grooming and decreased motivation in forced swim tests. Subsequently, proteomic analyses of brains from prenatally stressed pups, uncovered that the levels of Dihydropyrimidinase Like 2 (Dpysl2) were significantly reduced in samples containing prefrontal cortex and hippocampus crude protein extracts (Lee et al. 2015) .

Dpysl2 is a member of the collapsin response mediator protein (CRMP) family. In humans, the gene coding for DPYSL2 is mapped to chromosome 8p21 in humans, a chromosomal region associated with increased susceptibility to schizophrenia (Nakata et al. 2003; Zhao et al. 2006) . Dpysl2/CRMP2 is highly expressed in the developing nervous system and has been associated with microtubule stability, neuronal polarity, axonal outgrowth, and the modulation of synapse formation (Goshima et al. 1995; Inagaki et al. 2001; Yoshimura et al. 2005; Yamashita et al. 2012) .

To better understand why Dpysl2 is reduced in the brains of organisms subjected to prenatal stress, we studied UNC-33 , the *C. elegans* ortholog of human Dpysl2. Similarly to its vertebrate counterpart, UNC-33 is enriched in the nematode nervous system and plays an important role in neuronal development (Tsuboi et al. 2005) . Mutations in the *unc-33* gene result in uncoordinated locomotion, defects in axonal outgrowth, and mislocalization of axonal and dendritic proteins (Tsuboi et al. 2005; Maniar et al. 2011) . Furthermore, analysis of the effect of stressors on *unc-33* mutants showed that nematodes exposed to high temperature or bacterial infection during embryonic and early larval development suppress the activity of the *unc-33* promoter in neurons (Gonzalez-Garcia et al. 2022). Interestingly, the combination of these stressors did not show an additive effect in repressing the *unc-33* promoter in neurons, suggesting that stressors such as high temperature and bacterial infection may be acting using the same molecular pathway to repress neuronal expression of *unc-33* .

Herein, we introduced antipsychotic medications that include clozapine, olanzapine, and trifluoperazine to *C. elegans* grown at high temperatures to test two different phenomena: the activity of the *unc-33* promoter and lifespan. Typical antipsychotic drugs such as trifluoperazine act in humans by robustly blocking D2 dopamine receptors, while atypical antipsychotic drugs, including clozapine and olanzapine, additionally show high affinity for 5-HT _2A_ serotonin receptors (Meltzer and Gadaleta, 2021) . Of note, *C. elegans* has been shown to respond to these drugs through D2-like receptors and the 5-HT2-like receptor SER-1 . However, the developmental effects of antipsychotics in *C. elegans* may involve additional mechanisms; trifluoperazine, in particular, was shown to also act via inhibition of calmodulin signaling (Donahoe et al. 2006). Given this and other evidence indicating that the pharmacological activity of these drugs is likely complex, we wanted to see if these antipsychotic drugs act in *C. elegans* by regulating the expression of *unc-33* . We hypothesized that introducing antipsychotic medications will relieve the heat-induced suppression of the *unc-33* promoter in neurons and restore lifespan to normal levels. To test this hypothesis, *C. elegans* hermaphrodites were synchronized and incubated at 25°C to induce stress either in the presence or absence of each antipsychotic medication. Consistent with previously reported findings, imaging of *unc-33* promoter activity via a GFP-reporter showed that *C. elegans* grown at 16℃ had higher neuronal expression of GFP, while *C. elegans* grown at 25℃ had higher glial expression of GFP (Gonzalez-Garcia et al. 2022). Building from this, we performed experiments to test if exposure to antipsychotic drugs used to treat schizophrenia could restore neuronal expression of *unc-33* at 25℃ *.* Results from this assay indicate that the neuronal GFP expression driven by the *unc-33* promoter in heat-exposed nematodes treated with antipsychotic drugs was not significantly different from DMSO-treated *C. elegans.* Specifically, animals treated with DMSO had a ratio of neuronal/glial fluorescence of 0.70, while animals treated with clozapine, olanzapine, and trifluoperazine had ratios of 0.92, 0.79, and 0.76, respectively. These results suggest that clozapine, olanzapine, and trifluoperazine are unlikely to act via the regulation of the *unc-33* promoter in *C. elegans* .

To test whether the antipsychotic drugs would restore the nematodes' lifespan to normal levels, a lifespan assay was performed and monitored every day until all nematodes died. Contrary to our hypothesis that the antipsychotic drugs would restore lifespan to normal levels, we saw a decrease in survival in the presence of antipsychotic medication compared to worms exposed to solvent alone. Lifespan results indicate that the median survival of animals in DMSO was 12 days, while median survival in antipsychotic drugs clozapine, olanzapine, and trifluoperazine were 9.5, 9.5, and 11, respectively. Statistical analysis of lifespan curves denoted a significant difference between DMSO-treated animals and clozapine or olanzapine-treated animals. However, when comparing survival between nematodes exposed to DMSO and nematodes exposed to trifluoperazine, there was borderline significance, with the Mantel-Cox post hoc test showing a p-value of 0.0495. Taken together, this data demonstrates that exposure to antipsychotic medications did not restore lifespan to normal levels, and in fact, clozapine and olanzapine had a detrimental effect.

In conclusion, our findings demonstrate that the *C. elegans* *unc-33* promoter may be insensitive to antipsychotic drugs. In humans, genomic analysis of the 5'UTR sequences of *DPYSL2* uncovered a 5'-terminal oligopyrimidine (5'-TOP) tract that is regulated by the mTOR pathway (Pham et al., 2016) . Analysis of a schizophrenia-associated polymorphic CT-dinucleotide repeat (DNR) in the 5'-TOP tract of *DPYSL2* showed weaker binding of mTOR effectors and consequentially lower translation of *DPYSL2 ,* connecting DPYSL2 to schizophrenia and external environmental factors (Pham et al. 2016, Izumi et al., 2022) . More importantly, cells treated with the antipsychotics thioridazine, trifluoperazine, and prochlorperazine had the opposite transcriptome signature as those containing the schizophrenia-associated DNR allele (Pham et al. 2016) . Preliminary examination of the 5' UTR of *unc-33* does not identify 5'-TOP oligopyrimidines, possibly explaining the lack of effect seen in *C. elegans* treated with the antipsychotic drugs clozapine, olanzapine and trifluoperazine.

Significantly, while in contrast to our initial hypothesis, the observation of the decreased lifespan as a result of treatments with antipsychotic drugs is consistent with existing literature. In fact, Weeks et. al (2010) demonstrated not only that these same three drugs decrease lifespan, but also that this was dependent on the activation of Akt signaling by DAF-2 . Additionally, results published by Zarse and Ristow (2008) found that serotonin antagonist-type drugs, like those used in our study, do not extend the lifespan of *C. elegans* , while, Jiang and colleagues showed a decrease in *C. elegans* lifespan following mutation or inhibition of dopamine D2-like receptors (D2R). It is important to note that the mechanisms of action of trifluoperazine, clozapine, and olanzapine in *C. elegans* may not be limited to D2R and SER-1 , and that the findings presented here may be in part a consequence of the effects of these drugs on other receptors as well as functions for dopamine and serotonin outside of the regulation of DPYSL2 . However, they are consistent with previous data both characterizing these drugs specifically and other studies investigating their potential mechanisms. Thus, we conclude that our findings align with results indicating that antipsychotic drugs are not effective in producing an extension of lifespan, perhaps due to the absence of the requisite regulation of DPYSL2 by mTOR in *C. elegans* .

## Methods

*Synchronization of nematodes*

To synchronize nematodes to an L3 stage, we followed the protocol previously described by Garcia-Gonzalez et al (2022). In brief; synchronized embryos were harvested from adult gravid hermaphrodites after alkaline bleach treatment. These embryos were washed three times with M9 buffer and plated onto NGM plates containing *E. coli* OP50 . The L3 stage was reached after incubating harvested embryos at 16℃ for 48 hours or 25℃ for 24 hours.

*Preparation of Antipsychotic Drugs*

Clozapine, olanzapine, and trifluoperazine were purchased from Sigma-Aldrich. To prepare working solutions with these antipsychotic drugs, we first dissolved clozapine, olanzapine, and trifluoperazine in DMSO to obtain stock solutions of 160 mM, 160 mM, and 80 mM, respectively. Next, the stock solutions were diluted 20 times using 1.7 mM acetic acid to obtain working solutions of 8 mM clozapine, 8 mM olanzapine, and 4 mM trifluoperazine. The DMSO working solution for control treatments was prepared by diluting DMSO 20 times with 1.7 mM acetic acid. Last, for nematode treatments, 200 μL of the corresponding working solutions were used to impregnate NGM plates containing *E. coli* OP50 bacteria, resulting in the following final concentrations: 0.1% DMSO, 160 μM clozapine in 0.1%DMSO, 160 μM olanzapine in 0.1% DMSO, and 80 μM trifluoperazine in 0.1% DMSO.

*Analysis of the activity of the unc-33 promoter via fluorescence microscopy*

To examine the levels of activity of the *unc-33* promoter, we assessed the fluorescence of the GFP reporter using the protocol described by Garcia-Gonzalez et al. (2022). The heads of L3 nematodes were imaged using an Olympus Fluoview Confocal Laser Scanning Microscope (FV3000). Z-stacks were obtained from nematode heads, defined as the anterior tip of the animal to the nerve ring, using an oil immersion 20X objective (0.85 NA) and the optimal pinhole size of 111 µm. Images were produced by one-way line scanning with a Zoom of 4.43X, a sampling speed of 8 µs/pixel, integrating four frames, a laser transmissivity of 0.08%, and using the 500-600 nm detection wavelength range. The pinhole, ROI area, and laser power were unchanged in all images. To quantify fluorescence, the CellSens software was used to draw regions of interest (ROI) around glial cells and neurons. ROIs were converted to ‘Dynamic ROI over Z' and the Mean Maximum Intensity Value from images with subtracted backgrounds was plotted as the ratio of Neuronal/Glial fluorescence. The series analysis tool in Fluoview calculates the Mean Maximum Intensity Value by obtaining the average intensity of the brightest pixel in each ROI of every image in the series. Lastly, it is important to note that previous studies tailored to establish the best strategy to demonstrate and quantify the temperature-dependence of GFP expression driven by the *unc-33* promoter showed that the ratio of neuronal to glial fluorescence produced the most consistent numerical values of the phenomenon (Garcia-Gonzalez, et al (2022)). The measurement of just neuronal GFP fluorescence proved to be very variable; thus, having the amphid socket cell as a reference was very useful. The GFP expression in neuronal processes near the amphid socket cell is negligible compared to that observed in cell bodies.

*Lifespan Assay*

The survival of nematodes was quantified by evaluating 20 synchronized young hermaphrodites per condition and assessing their survival every 24 hours until death. In short, synchronized nematodes were cultivated on NGM plates containing *E. coli* OP50 bacteria and impregnated with DMSO, 160μM clozapine, 160μM olanzapine, or 80μM trifluoperazine at 16℃ or 25℃. Once they reached the L4 stage, 20 nematodes were transferred onto new plates containing the corresponding antipsychotic drug and recorded as Day 0 of adulthood. The survival of nematodes was monitored daily, and living animals were transferred to new plates with *E. coli* OP50 and antipsychotic drugs every three days until all remaining animals died. Newly born animals and eggs were removed from plates on non-transfer days to ensure only original animals were counted. Survival was recorded as a percent ratio of living to dead nematodes in 3 biological replicas per condition.

*Statistics*

Statistical analyses were performed using GraphPad Prism version 9.4.0. The error bars represent standard deviations and statistical differences plotted as *p<0.05,**p<0.01, ***p<0.001, *p<0.0001. Statistical analysis for neuronal/glial fluorescence, as shown in Figure 1A, was analyzed by performing a Shapiro -Wilk normality test followed by a Kruskal-Wallis test and Dunn's multiple comparison test. The survival assay demonstrated in Figure 1B was analyzed by arranging the data according to Kaplan Meier survival analysis followed by Mantel -Cox post hoc and the Logrank test for trend.

## Reagents

*Strains*

OH438 *unc-4 ( e120 )* II; *otIs117* [ *unc-33p* ::GFP + *unc-4* (+)]. IV strain was obtained from *C. elegans* Genetics Center.
